# Ivonescimab for EGFR-mutant lung adenosquamous carcinoma after multiline therapy: A case report

**DOI:** 10.3389/fonc.2025.1707096

**Published:** 2025-11-26

**Authors:** Xiaohua Pan, Jianya Zhang, Chao Ye, Wanhui Zhu, Wenshu Qu

**Affiliations:** 1 School of Basic Medicine and Clinical Pharmacy, China Pharmaceutical University, Nanjing, China; 2 The First Affiliated Hospital of China Pharmaceutical University, Nanjing Tianyinshan Hospital, Nanjing, China

**Keywords:** EGFR-mutant, adenosquamous carcinoma, ivonescimab, NSCLC, multiline therapy

## Abstract

This case report describes a 51-year-old male with advanced Epidermal Growth Factor Receptor (EGFR) mutant (p.T790M and p.L858R) lung adenosquamous carcinoma who achieved a rapid partial response (PR) to ivonescimab monotherapy following progression on multi-line therapies, including third-generation EGFR-tyrosine kinase inhibitors (EGFR-TKIs), platinum-based chemotherapy, anti-angiogenic therapy, and immune checkpoint inhibitors. Despite initial responses to first-line firmonertinib-based combination therapy, progression-free survival (PFS) 13 months, the patient developed sequential resistance to subsequent regimens, including liver/brain metastases and treatment-related toxicities. After fourth-line therapy failure and severe intolerance to albumin-bound paclitaxel and bevacizumab, two cycles of ivonescimab—a first-in-class programmed cell death protein receptor-1 (PD-1)/vascular endothelial growth factor-A (VEGF-A) bispecific antibody—induced significant regression of pulmonary target lesions (PR), sustained over six cycles with minimal toxicity. This case highlights ivonescimab’s dual-mechanism potential to overcome resistance in EGFR-mutant Non–small cell lung cancer (NSCLC) by concurrently alleviating PD-1-mediated immunosuppression and VEGF-driven angiogenesis. The observed efficacy in a low PD-L1 expression, tumor proportion score (TPS) 5%, tumor protein 53 (TP53)-co-mutated, and adenosquamous histology context aligns with prior clinical trial data (HARMONi-A/2), suggesting broad applicability across heterogeneous subgroups. While the rapid PR and favorable safety profile are promising, longer follow-up is required to assess durability and survival benefits. These findings underscore the need for further investigation of bispecific antibodies in precision oncology paradigms for multi-refractory EGFR-driven NSCLC.

## Introduction

According to the latest cancer data from the International Agency for Research on Cancer (IARC) in 2022, the incidence rate and mortality rate of lung cancer among various types of cancer rank first globally (1). NSCLC comprises approximately 80% to 85% of all lung cancers, with EGFR mutation rates as high as 51.4% in the Asian NSCLC population (2, 3).

Third-generation EGFR-TKIs are approved as the first-line treatment for patients with advanced EGFR-mutant NSCLC; however acquired resistance is also inevitable in such patients, and the options for posterior-line therapy remain limited (4–7). It is difficult to benefit from immune monotherapy after resistance in patients with EGFR-mutant, and combination anti-vascular therapy may be potentially favorable (8). Ivonescimab (AK112/SMT112) is the first humanized, bispecific monoclonal antibody specifically binding human VEGF-A and PD-1. Based on the findings of the HARMONI-A study, the National Medical Products Administration (NMPA) approved ivonescimab, combined with chemotherapy, for the treatment of EGFR-mutated locally advanced or metastatic non-squamous NSCLC patients who have progressed after treatment with EGFR-TKIs, in May 2024 (9, 10).

This article reports a patient with EGFR-mutant who achieved PR in the short term with monotherapy with ivonescimab after multiline therapy progression in advanced lung adenosquamous carcinoma.

## Case presentation

A 51-year-old male with chronic hepatitis B virus (HBV) infection (20+ years, entecavir-treated) presented with cough and weight loss. Positron emission tomography-computed tomography (PET-CT) (September 7, 2022) revealed a left upper lobe mass (79 × 57 × 53 mm, FDG SUV_max_=12.00) with intrapulmonary metastases, left sixth posterior rib osteolysis (FDG SUV_max_=13.51), left hilar/mediastinal lymphadenopathy (FDG SUV_max_=4.78), and a hypodense hepatic lesion (FDG SUV_max_=7.79). Brain magnetic resonance imaging (MRI) confirmed no intracranial metastasis. On October 11, 2022, CT-guided needle biopsy of local soft tissue lesions in left rib, the pathological findings showed metastatic poorly differentiated adenocarcinoma with partially poorly differentiated squamous cell carcinoma. Gene panel testing identified EGFR (p.T790M and p.L858R) mutations, TP53 mutation, tumor mutational burden (TMB 1.25Muts/Mb), PD-L1(TPS 5%, CPS 7), microsatellite stable (MSS). First-line regimen therapy (from October 15, 2022 to May 20, 2023): 6 cycles of AP regimen (Pemetrexed 1.0 g, Carboplatin 500 mg) plus firmonertinib (80 mg) achieved PR. After that Single-agent Pemetrexed (1.0 g) was given for 3 cycles of maintenance treatment, while the oral firmonertinib (80 mg) was given continuously. The PFS under the first-line treatment was 13 months.

Subsequently, on October 20, 2023, the patient experienced progressive disease (PD), with an increase in the size of the left upper lobe target lesion by 20%, with emergence multiple metastases within the liver. A CT-guided percutaneous lung biopsy was performed, followed by genetic analysis of the pathological tissue. The results confirmed the presence of EGFR L858R/T790M mutations and TP53 mutation. The identified mutation sites remained the same, but their abundance decreased compared to previous assessments. Second-line regimen therapy (from October 23, 2023 to March 12, 2024): 6 cycles of Paclitaxel Polymeric Micelles (300 mg) plus anlotinib (12 mg/day) based on continuous firmonertinib (80 mg), to evaluate the best efficacy stable disease (SD). Radiofrequency ablation for treatment of tumors in the right lobe of liver on November 24, 2023. Brain MRI on February 19, 2024, revealed a solitary metastatic lesion (2.9 × 2.5 cm) adjacent to the right lateral ventricle, while concurrent thoracoabdominal CT demonstrated stable pulmonary and hepatic lesions. To address central nervous system (CNS) progression, whole-brain radiotherapy (WBRT) (30 Gy/10 fractions) was initiated on February 23, 2024, followed by a local boost (25 Gy/5 fractions) to the right lateral ventricular lesion on March 8, 2024, per oligometastatic management guidelines. Concurrently, firmonertinib was escalated to 160 mg/day to enhance blood-brain barrier penetration, while anlotinib (12 mg/day) was maintained to sustain anti-angiogenic efficacy.

Post-radiotherapy imaging (April 7, 2024) demonstrated a persistent left upper lobe soft tissue mass (94 × 53 mm) on thoracic CT and a heterogeneous right basal ganglia lesion (38 × 32 × 41 mm) on brain MRI. Whole-body PET-CT (April 11, 2024) demonstrated mixed therapeutic responses: (1) Hypermetabolic foci in the left upper lung mass (FDG SUV_max_=18.54), mediastinal lymph nodes (FDG SUV_max_=3.57), hepatic segment VII hypodense nodule (FDG SUV_max_=7.2), and T6 vertebral osteolytic destruction (FDG SUV_max_=21.12), indicating residual tumor activity; (2) Complete metabolic suppression (FDG SUV_max_=2.1) of the left sixth posterior rib metastasis, confirming treatment efficacy; (3) A right cerebral lesion exhibiting post-radiation hemorrhagic necrosis and perilesional edema on MRI, with absent FDG avidity, consistent with inactive inflammatory changes. Meanwhile, the patient had paroxysmal irritant cough hemoptysis, coughed out necrosis, and lost 5 kg weight in 6 weeks. Poorly differentiated carcinoma confirmed by pathology and immunohistochemistry of necrosis. The genetic testing sent for indication that the specimen was unqualified. Comprehensive evaluation of the pathogenetic condition Re-PD. Thus, stopped oral firmonertinib and anlotinib.

Third-line regimen therapy (from April 16, 2024 to June 11, 2024): 3 cycles of AP regimen (Pemetrexed 1.0 g, Cisplatin 120 mg), evaluate the best efficacy SD. During the course of treatment (June 6, 2024), the patient developed postural low back pain, which significantly impaired the quality of life of the patient. A re-evaluation with magnetic resonance imaging (MRI) indicated an association with vertebral metastasis. The pain was subsequently alleviated following a combined approach of palliative radiotherapy.

**Figure 1:**
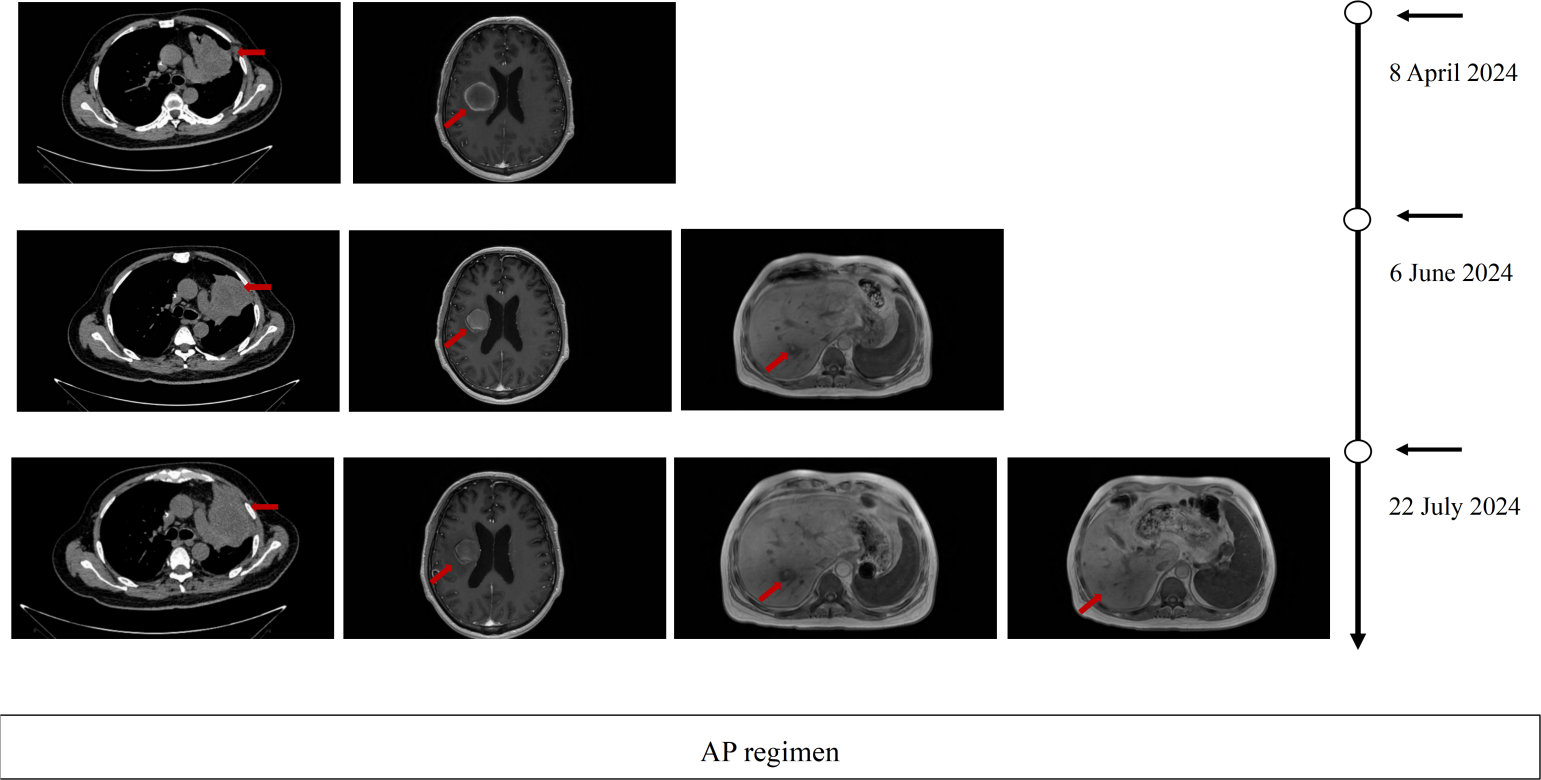
Imaging evaluation during third-line. Therapy Serial thoracic CT, brain MRI, and hepatic MRI at baseline, after 2 cycles, and after 3 cycles of third-line therapy PD per RECIST 1.1 criteria.

**Figure 2:**
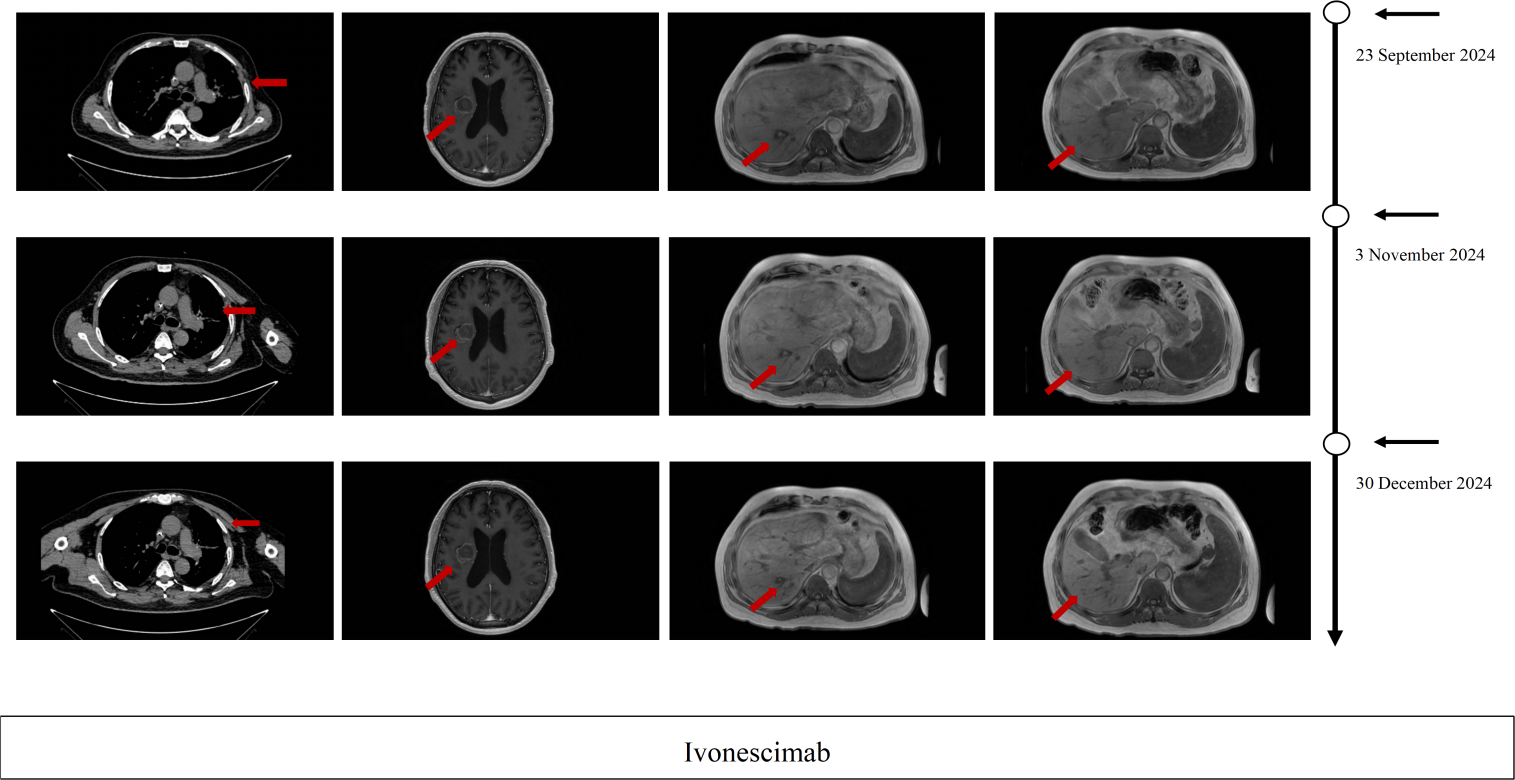
Imaging evaluation during ivonescimab. Therapy Serial thoracic CT, brain MRI, and hepatic MRI after 2 cycles, after 4 cycles and after 6cycles of ivonescimab therapy PR per RECIST 1.1 criteria.

On July 2024, Pulmonary CT imaging revealed that the target lesion in the left lung had enlarged compared to previous scans. Upper abdominal MRI indicated an increase in liver metastases from prior findings. A comprehensive assessment concluded that the disease had once again progressed to PD (**Figure 1**). On July 24, 2024, the patient underwent one cycle of fourth-line treatment with Albumin-bound Paclitaxel (200 mg) in combination with Bevacizumab (500 mg). After treatment, the patient experienced systemic fatigue (CTCAE grade 3), bone marrow suppression (CTCAE grade 3), and alopecia (CTCAE grade 2), among other side effects. The patient experienced significant intolerance to the treatment. On August 7, 2024, and August 29, 2024, the treatment was switched to monotherapy with ivonescimab for two cycles. After two cycles, follow-up imaging showed that the target lesion in the left lung had significantly reduced in size compared to previous scans, and the therapeutic efficacy was evaluated as PR. From September 24, 2024 to December 5, 2024, the patient continued ivonescimab treatment for an additional four cycles. After the fourth cycle, a follow-up chest CT and brain MRI on November 3, 2024, indicated further shrinkage of the target lesions, and the therapeutic efficacy was maintained as PR (**Figure 2**). It is noteworthy that during the course of ivonescimab therapy, the patient did not present with hemoptysis or expectoration of necrotic material, only a mild elevation in blood pressure was observed.

## Discussion

In this case report, we describe a patient with EGFR-mutated lung adenosquamous carcinoma who exhibited persistent tumor progression despite multiple lines of therapy following resistance to third-generation EGFR-TKIs treatment. Notably, after two cycles of ivonescimab monotherapy, the target lesion achieved a PR, demonstrating a remarkable and clinically meaningful response. This outcome highlights the potential efficacy of ivonescimab in managing advanced EGFR-mutant NSCLC cases refractory to standard therapies.

TKIs have dramatically changed the clinical prospects of patients with non-small cell lung cancer harboring EGFR-activating mutations. Despite prolonged disease control and high tumor response rates, all patients eventually progress on EGFR-TKIs treatment (11). Patients with EGFR mutation-positive advanced NSCLC who develop resistance to TKIs often exhibit suboptimal clinical outcomes when treated with standard platinum-based chemotherapy. Moreover, monotherapy with immune checkpoint inhibitors (ICIs) has not shown a significant improvement in efficacy in this population. Given the limited efficacy of available subsequent therapies, this high-risk subgroup faces a critical unmet need, underscoring the imperative to develop novel strategies that overcome resistance and prolong survival (12, 13). The application of ICIs in patients with advanced EGFR mutation-positive NSCLC who have developed resistance to TKIs remains a subject of ongoing debate. However, emerging evidence suggests that vascular endothelial growth factor (VEGF) inhibitors may play a pivotal role in enhancing the therapeutic efficacy of PD-1/PD-L1 antibodies. This is achieved through the transformation of the immunosuppressive tumor microenvironment (TME) into an immunoreactive TME, which facilitates increased immune cell infiltration and activation. Such mechanisms hold significant promise for overcoming resistance and improving clinical outcomes in this challenging patient population (14). Combination therapy involving anti-angiogenic agents and ICIs has demonstrated significant clinical efficacy in patients with NSCLC, irrespective of PD-L1 expression status and EGFR mutation status. However, this therapeutic approach is also associated with an increased risk of various adverse events, highlighting the need for careful patient selection and monitoring to balance efficacy and safety (15, 16).

Ivonescimab is a first-in-class humanized tetravalent bispecific antibody engineered to exert dual mechanisms of action. It competitively inhibits the binding of PD-1 to its ligand PD-L1, thereby reversing PD-1/PD-L1-mediated immunosuppression, while concurrently blocking VEGF-A interaction with VEGFR2 to suppress tumor angiogenesis within the tumor microenvironment. This bifunctional targeting strategy integrates immune checkpoint blockade with anti-angiogenic effects, offering a synergistic therapeutic approach to counteract both immune evasion and tumor vascularization (17). Ivonescimab is a tetravalent, symmetric bispecific antibody capable of high-affinity binding to both PD-1 and VEGF-A. Within the tumor microenvironment, its interaction with VEGF-A dimers enhances PD-1 affinity, thereby more precisely reversing immunosuppression and promoting vascular normalization. Preclinical studies have shown that this VEGF-dependent interaction facilitates preferential accumulation and prolonged retention of ivonescimab in VEGF-rich tumor tissues. By simultaneously blocking PD-L1 and VEGF pathways, ivonescimab achieves synergistic antitumor activity with greater target specificity while minimizing overlapping toxicities associated with dual-agent regimens. Conventional combinations of immunotherapy and anti-angiogenic agents (e.g., PD-1 inhibitors with bevacizumab or TKIs) often suffer from pharmacokinetic incompatibilities and additive toxicities, such as TKI-induced hepatotoxicity and hand–foot syndrome. As a single-molecule agent, ivonescimab eliminates Fc-mediated ADCC and CDC through Fc engineering, thereby reducing immune-related adverse events and offering improved pharmacokinetic properties with a more manageable safety profile (14, 18). Such dual-pathway inhibition positions ivonescimab as a compelling candidate for malignancies characterized by immunosuppressive microenvironments and aberrant angiogenic signaling, with potential implications for overcoming resistance to monotherapy regimens. ivonescimab has demonstrated promising antitumor activity and a manageable safety profile in patients with EGFR-mutant advanced NSCLC who had progressed on prior EGFR-TKIs therapy, as well as in those with advanced NSCLC refractory to platinum-based chemotherapy, PD-1/PD-L1 inhibitors, and antiangiogenic therapies (14, 19). The HARMONi-A/2 study demonstrated consistent and significant clinical benefits across all pre-specified subgroups with ivonescimab, irrespective of treatment regimen (chemotherapy-containing or chemotherapy-free). Notably, robust positive outcomes were observed in patients stratified by PD-L1 expression levels (1–49% or ≥50%), histology (non-squamous or squamous), baseline central nervous system (CNS) metastases, liver metastases, or co-existing driver gene alterations. Compared with pembrolizumab, patients treated with ivonescimab achieved a significantly longer median PFS. These findings highlight the broad applicability of ivonescimab in diverse clinical scenarios (20, 21). Furthermore, the HARMONi-5 trial confirmed the reliable safety profile and favorable efficacy of ivonescimab monotherapy in advanced NSCLC (22).

In the present case, a patient with EGFR-mutated NSCLC who developed resistance to third-generation TKIs and subsequent disease progression despite chemotherapy and antiangiogenic therapy achieved marked regression of pulmonary target lesions with ivonescimab monotherapy, accompanied by minimal treatment-related toxicity. This aligns with prior clinical evidence, suggesting that ivonescimab may serve as a viable therapeutic option for patients exhausted by standard therapies, particularly in the context of complex resistance mechanisms and multi-line treatment failure.

## Conclusion

In this study, we present a case of advanced EGFR-mutated lung adenosquamous carcinoma in which the patient achieved a partial response (PR) within a short treatment duration following monotherapy with ivonescimab, a PD-1/VEGF-A bispecific monoclonal antibody, after progression on multiple lines of therapy. This case highlights the potential of dual-targeting antibodies as a novel therapeutic strategy for patients with EGFR-TKI-resistant advanced NSCLC who have exhausted standard treatment options. However, due to the limited follow-up duration in this study, the long-term clinical benefits, including overall survival (OS) improvement, remain undetermined. Further studies with extended follow-up and larger cohorts are warranted to validate these findings and elucidate the durability of response.

## Data Availability

The original contributions presented in the study are included in the article/supplementary material. Further inquiries can be directed to the corresponding author.
